# Cognitive Resources in Working Memory: Domain-Specific or General?

**DOI:** 10.3390/bs12110459

**Published:** 2022-11-17

**Authors:** Anna Izmalkova, Artem Barmin, Boris B. Velichkovsky, Gerda Prutko, Igor Chistyakov

**Affiliations:** 1 Laboratory for Cognitive Studies of Communication, Moscow State Linguistic University, 119034 Moscow, Russia; 2 Centre for Cognition and Decision Making, Insitute for Cognitive Neuroscience, National Research University Higher School of Economics, 101000 Moscow, Russia; 3 Faculty of Psychology, Lomonosov Moscow State University,125009 Moscow, Russia

**Keywords:** working memory, domain-specific resources, domain-general resources, region of direct access, activated long-term memory, cognitive load

## Abstract

An experiment in the dual-task paradigm was carried out to explore the nature of domain-specific and domain-general resource distribution in working memory. The subjects (N = 32) performed symmetry span and letter reading span tasks under visuospatial (tapping) and verbal (articulatory suppression) cognitive load. The effects of task type and cognitive load modality were analyzed. The results are described within the concentric model framework: significant distinctions in relative accuracy under visuospatial and verbal cognitive load in visuospatial and verbal tasks were observed when N elements in the set exceeded the region of direct access capacity, while no such effect was observed for 2–3 element sets. This is attributed to domain-general resources in the region of direct access, and domain-specific resources in the activated long-term memory. We also found evidence for the asymmetric distribution of visuospatial and verbal working memory resources in that the verbal component is more susceptible to cognitive load.

## 1. Introduction

Working memory (WM) is a system of cognitive processes that store and process information that is currently being used in a cognitive task [1,2]. WM is responsible for maintaining information in an activated state, searching and selecting information from long-term memory, transforming retained cognitive representations, and saving intermediate processing results [3,4]. The significant role of WM is highlighted by strong interrelation with higher-order cognitive functions, such as reading comprehension [5] and general intelligence [6].

To date, the most influential models of WM are the multi-component model and the activation-based models. The multi-component model, proposed in the seminal work of A. Baddeley and G. Hitch [7], have gained substantial empirical evidence [8]. According to the model, WM encompasses two unimodal storage systems (“slave systems”), responsible for the transient storage of domain-specific information: a phonological loop and a visuospatial sketchpad; a central executive, responsible for the control of attention and information processing; and, according to a later revision of the model, a further component—a multimodal store capable of integrating information into unitary episodic representations, termed episodic buffer [9]. 

While the multi-component model emphasizes modality-based WM components, the activation-based models distinguish various representational states of the information held in WM [10]. For instance, in the concentric model, proposed by K. Oberauer, three states of representations in WM are distinguished: the focus of attention (elements, that are already selected for whatever cognitive operation is set up), the capacity limited region of direct access (elements that form the selection set to be retrieved), and the activated part of long-term memory (elements that are held available in the background) [11]. The model is based on empirical evidence of WM span and word updating tasks [4,12]. It must be noted that the three regions are not regarded as separate systems (by contrast with the multi-component model), but are supposed to be functionally different states of representations in WM. Moreover, the concentric model is not in total discord with the multi-component model, since the findings can be accommodated within alternative theoretical frameworks (for instance, by assuming that the active sets are held in the central executive, whereas the passive sets are held in the phonological loop or in the visuospatial sketchpad), however, they are not predicted by them [11].

To date, no consensus has been achieved concerning the distribution of cognitive resources within WM components. The approaches to the interplay of domain-specific components in WM range from assumptions of their independence [13] to acknowledging their interplay [14]. A possible solution to the problem is acknowledging the domain-general nature of central components and the domain-specific nature of peripheral components [15]. However, the features of this interplay are not yet determined.

According to the premises of the concentric model, it is the distribution of cognitive resources that can account for the interplay of the visual-spatial and verbal components in WM. Therefore, the key challenge is determining the role of domain-specific and domain-general resources in WM. For instance, M. Kane et al. obtained evidence in favor of the domain-general view of WM capacity, since WM span measures were determined by executive-attention processes, while domain-specific storage and rehearsal processes underlie domain-specific aspects of complex cognition [16]. It is thus assumed that domain-general resources provide for domain-specific resources. The studies of WM tasks performance under cognitive load, conducted by E. Vergauwe et al., support this view: a decrease in WM performance in intense cognitive load, regardless of the mismatch of the material modality in the tasks performed, can also confirm the existence of a central pool of general resources in WM [17]. In later research, an asymmetry between verbal and spatial information was observed: spatial information can solely rely on attention for its maintenance while verbal information can also rely on a domain-specific maintenance mechanism independent from attention [18]. Therefore, the assumption of domain-general and domain-specific resources providing for WM components (as distinguished in the concentric model) according to the task at hand, seems appropriate. We claim that the relative contribution of domain-general and domain-specific resources depends on internal (energetic, motivational, and strategic) and external (requirements of the task) conditions.

The most influential method for assessing WM span is the dual-task paradigm, proposed by A. Baddeley and G. Hitch: WM retention task (e.g., memorizing digits) is performed along with an additional information processing task (e.g., solving math operations) [7,19]. Methodologically close to the dual-task paradigm is the loading paradigm, which is applied, for instance, in the processing efficiency theory [7,20]: the primary task (e.g., verbal reasoning) is performed concurrently with a secondary task (e.g., spatial tapping). The concurrent performance of two tasks provides two measures of cognitive load: the extent to which the primary task performance is affected by the secondary task, and the performance of the secondary task itself (see, e.g., Esmaeili Bijarsari, 2021 [21] for the taxonomy of dual-tasks). 

This study is aimed at investigating the process of dynamic distribution of WM resources between visuospatial and verbal components under visuospatial and verbal cognitive load. The dual-task paradigm, proposed by A. Baddeley and G. Hitch was applied: WM retention task was performed along with an additional information processing task [7]. 

Apart from the traditional dual-task with primary and secondary tasks, in accordance with the “loading” paradigm [20], an additional cognitive load was implemented. Thus, the primary working memory task was being performed concurrently with two secondary tasks. The dual-task and the load task addressed either the visuospatial or the verbal WM component. Thus, the effect of the task and cognitive load modality on information retention in WM was tested.

## 2. Materials and Methods

### 2.1. Participants

The research was conducted on a sample of 32 participants, with ages ranging between 19–23 years (median = 20), 27 females and five males. The subjects were recruited at Moscow State Linguistic University. All of them were second-year bachelor students, receiving course credit for their participation. Informed consent was obtained from all subjects involved in the study.

### 2.2. Measures

**Figure 1:**
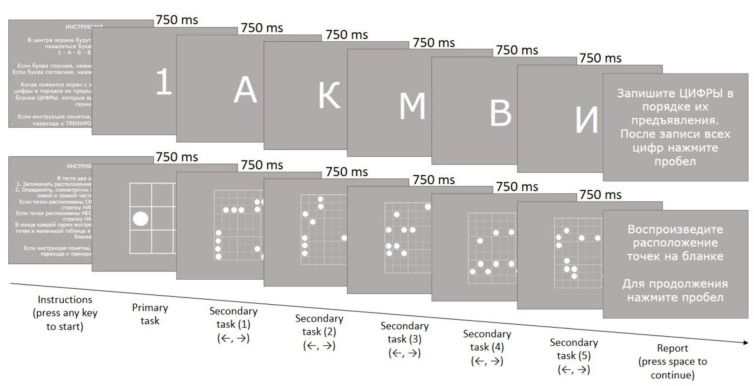
Experimental sequence of stimuli presentation during the letter reading span and symmetry span tasks.

The letter reading span task was aimed at assessing verbal WM capacities [22]. The participants were presented with a sequence of stimuli: digits and letters (see Figure 1). Each sub-trial consisted of a digit (from 1 to 9) and five Russian letters. Each stimulus was displayed in the center of the laptop screen for 0.75 s. The number of sub-trials in 10 consecutive trials was 2-3-4-5-6-6-5-4-3-2. The participants were asked to remember the digits and recall them in the correct sequence using the form (primary task), while at the same time trying to indicate whether each letter was a vowel or a consonant by pressing the corresponding key on the keyboard (“←”-vowel, “→”-consonant) (secondary task).

The symmetry span task was aimed at assessing visuospatial WM capacities [23]. The participants were presented with a sequence of stimuli: a 3 × 3 grid containing a single circle and five 9 × 9 grids containing 16 circles each (see Figure 1). Each stimulus was displayed in the center of the laptop screen for 0.75 s. The number of sub-trials in 10 consecutive trials was 2-3-4-5-6-6-5-4-3-2. The participants were asked to remember the circle positions in the 3 × 3 grid and recall them in the correct sequence using the form (primary task), while at the same time trying to indicate whether each circle position in the 9 × 9 grids was symmetrical by pressing the corresponding key on the keyboard (“←”-asymmetrical, “→”-symmetrical) (secondary task).

The duration of the longest sub-trial (containing six elements) was 27 s, to fall within the assumed working memory time limits. Subjects recalled the stimuli of the primary task after each trial. Thus, the subjects had to store from two to six elements in the working memory.

### 2.3. Apparatus and Stimuli

The experiment was conducted using a laptop. The laptop device had the following configurations: Huawei MateBook D 15 (53012KRC), 15.6″ (1920 × 1080), Intel Core i5-10210U (1.6 GHz). Stimuli were displayed with Psychopy v. 22.1.1. Participants’ answers were registered with a standard keyboard and mouse.

### 2.4. Cognitive Load

Two types of cognitive load were used: verbal and visuospatial. The additional task (articulatory suppression and spatial tapping) was intended to tax either visuospatial sketchpad or phonological loop (already engaged in the resource-consuming dual-task).

Verbal cognitive load implies the activity of the verbal WM. The participants were instructed to repeat, as fast and as accurate as possible, four digits while performing WM span tasks. The sequence for repetition was 3-1-2-4. The model rate of repetition was given before every trial (2 s).

Visuospatial cognitive load implies the activity of the visuospatial WM. The participants were instructed to repeat, as fast and as accurate as possible, four mouse button clicks, while performing WM span tasks. The sequence of mouse clicks for repetition was left-right-right-left. The model rate of mouse clicks was given before every trial (2 s).

The task we used was modeled upon typical visuospatial load tasks, where subjects were supposed to tap a given pattern at a certain rate [20]. However, following M. Emerson and A. Miyake, who used foot tapping task when finger tapping interfered with the motor requirements involved in writing down numerical answers [19], we modified the traditional spatial tapping task in order to make our experiment design less obtrusive.

### 2.5. Procedure

**Figure 2:**
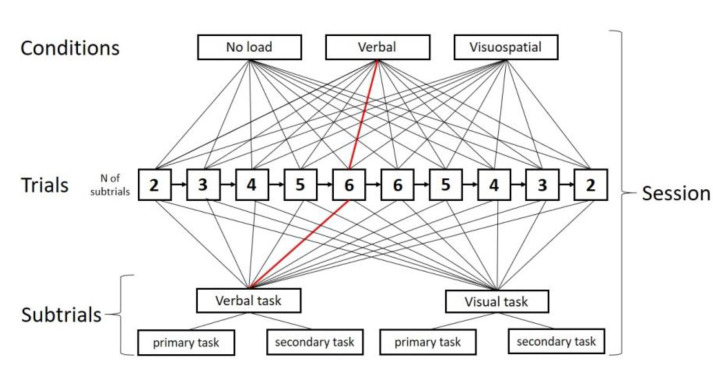
The experimental session scheme. The marked line is an example of a random trial that included six verbal sub-trials in verbal cognitive load condition.

The experiment was conducted in a quiet laboratory room. Before the experiment, the participants were asked to read the instructions and complete the training segments containing two WM span task trials (of each WM span task type). Each participant performed a sequence of trials. Symmetry and letter reading span tasks were given in no load condition first. Then, the task type and the load type varied cross-individually according to the unbalanced Latin square scheme. Thus, each participant performed six experimental sessions, 60 trials, and 240 sub-trials (see Figure 2). Each trial consisted of 2–6 sub—trials (the number of sub-trials was 2-3-4-5-6-6-5-4-3-2 for 10 consecutive trials). Each sub-trial consisted of a primary task (remembering) and a secondary task (processing), with additional load imposed in verbal and visuospatial load conditions.

### 2.6. Data Analysis

The normality distribution of the variables was evaluated with the Kolmogorov–Smirnov test. Data pertaining to the two experimental conditions (verbal cognitive load, visuospatial cognitive load) in each group (letter reading span task, symmetry span task) were analyzed using the Mann–Whitney non-parametric U-test. The two-way ANOVA was used to estimate how the mean of quantitative variable changes according to the levels of two independent variables: WM task (levels: letter reading span task, symmetry span task), cognitive load type (levels: verbal cognitive load, visuospatial cognitive load). We used the Wilcoxon non-parametric test as a follow-up to the two-way ANOVA to perform pairwise comparisons. All analyses were conducted by IBM SPSS Statistics for Windows, Version 22.0.

## 3. Results

The proportion of correctly recalled elements in each experimental session (including from two to six trials) was calculated to determine the WM accuracy. Accuracy scores as a result of counting correctly recalled elements are typical for studies using WM span tasks: reading span task [24], and symmetry span task [25]. The results ranged from 0 (no digit was recalled) to 1 (all digits were correctly recalled). Shifts in the proportion of correctly recalled elements in cognitive load conditions were included in the data processing. To keep the results commensurate across the conditions, we subtracted the no-load condition results from the cognitive load condition results in each experimental session. The resulting coefficients were designated as Relative Accuracy index (RA). The resulting coefficients were compared in two experimental conditions depending on the type of cognitive load. No significant distinctions were found in relative accuracy under verbal cognitive load (M = −0.13, SD = 0.44) and visuospatial cognitive load (M = −0.11, SD = 0.43), Z = −0.35, *p* > 0.05 in all the trials.

### 3.1. Mann–Whitney U-Test Results

The Kolmogorov–Smirnov test was used before the Mann–Whitney U-test. The results showed that the RA in each experimental condition did not conform to a normal distribution (Z varied from 0.167 to 0.284; *p* < 0.01).

Letter Reading Span task. Significant RA difference was found in two experimental conditions depending on the type of cognitive load (Z = −2.8, *p* < 0.01). The letter reading span task performed better in the visuospatial cognitive load condition (M = −0.15, SD = 0.39), than in the verbal cognitive load condition (M = −0.23, SD = 0.40). 

Symmetry Span task. There was no significant RA difference between the visuospatial cognitive load condition (M = −0.07, SD = 0.46) and the verbal cognitive load condition (M = −0.03, SD = 0.48) (Z = −0.995, *p* > 0.05).

The results allow us to determine the contribution of the cognitive load type to the RA of WM span task performance. Thus, cognitive load type had a significant effect on the RA of the letter reading span task performance, while having no effect on the RA of the symmetry span task performance.

### 3.2. Data Splitting

**Figure 3:**
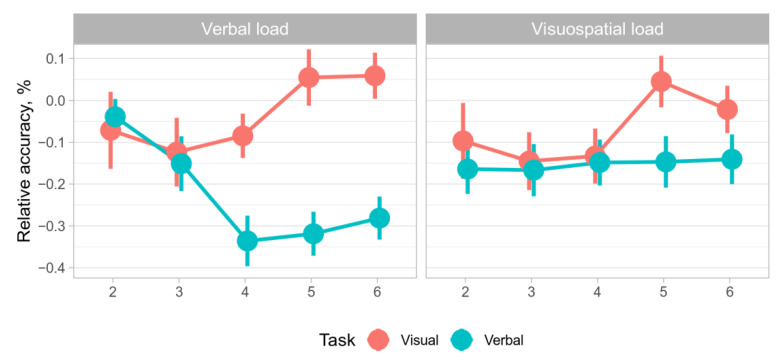
RA as a function of the number of items in the display.

In accordance with the concentric model framework, we split the data into two parts: trials with sets of 2–3 elements and trials with sets of 4–6 elements. Based on the tenets of the concentric model, 2–3 elements fall within the region of direct access, whereas 4–6 elements engage activated long-term memory [11]. This is in agreement with the relevant theory of short-term memory storage capacity [26]. As shown in Table 1, the three-way ANOVA analysis revealed significant interaction of three main factors, while only task type was significant as the main factor. The nature of said interaction is revealed in Figure 3, which demonstrates significant interactions between factors of Task and Load type: under visuospatial load, RA was not affected by the number of items in a verbal task, and even increased for visual task. The latter pattern is repeated for the visual task under visual load, while under verbal load RA drops significantly. This heavily implies that domain-specific resources are at play since limitations of general resources would have yielded similar patterns across different conditions, showing no significant interactions between Task and Load types.

### 3.3. Two-Way ANOVA Results

The Kolmogorov–Smirnov test was used before the two-way ANOVA. The results showed that the RA in each experimental condition did not conform to a normal distribution (Z varied from 0.119 to 0.368; *p* < 0.01). 

**Figure 4:**
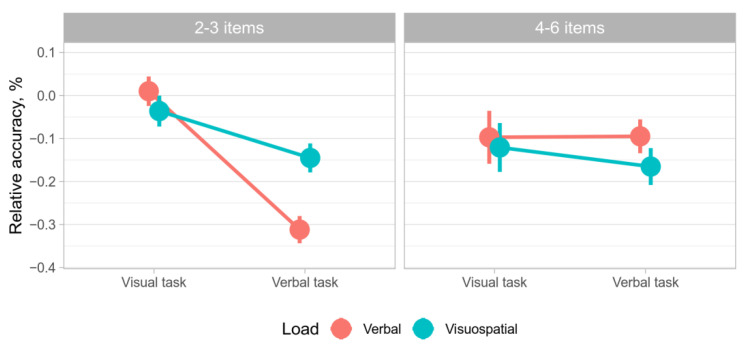
The two-way ANOVA results of the experimental sessions grouped by the number of items per trial.

Experimental sessions including 4–6 trials. A two-way ANOVA revealed a significant interaction between factors (F (1, 755) = 13.96, *p* < 0.01). The results also showed that the cognitive load type had a significant effect on the RA of the WM span task performance (F (1, 755) = 4.47, *p* < 0.05). The two-way ANOVA results are shown in Figure 4. The Wilcoxon non-parametric test results showed a statistically significant RA difference between cognitive load type levels (Z = −2.943, *p* < 0.01) and between WM span task levels (Z = −6.690, *p* < 0.01).

Experimental sessions including 2–3 trials. A two-way ANOVA didn’t reveal a significant interaction between factors (F (1, 504) = 0.3, *p* > 0.05). The results also showed that the cognitive load type had no effect on the RA of the WM span task performance (F (1, 504) = 1.21, *p* > 0.05). The two-way ANOVA results are shown in Figure 2. The Wilcoxon non-parametric test results showed that there was no significant RA difference between cognitive load type levels (Z = −1.230, *p* > 0.05) and between the WM span task levels (Z = −0.332, *p* > 0.05).

Two-way ANOVA results of the experimental sessions including 4–6 trials showed that the RA of WM span task performance varied depending on cognitive load type. Pairwise comparisons showed the significant effect of both factors. The two-way ANOVA results of the experimental sessions including 2–3 trials showed that the RA of the WM span task performance did not vary depending on cognitive load type. Pairwise comparisons showed that there was no significant effect of both factors.

Summarizing this section, there is evidence that RA of letter reading span task performance is determined by the cognitive load. Contrastingly, the RA of symmetry span task performance is not susceptible to cognitive load. The RA of 2–3 sub-trial performance does not vary depending on cognitive load. However, The RA of 4–6 sub-trial performance is characterized by variability caused by the cognitive load.

## 4. Discussion

The effect of cognitive load modality on the relative accuracy of WM tasks performance was established: visuospatial cognitive load was associated with a higher relative accuracy score. This effect can be attributed to the depletion of cognitive resources involved in the verbal WM component under verbal cognitive load. Similar results were obtained in the study of articulatory suppression [27]: an additional task of articulating irrelevant verbal material (thus inhibiting inner speech) brought about a decrease in verbal information retention in WM. However, this statement is justified only assuming that the verbal WM component is less sensitive to visuospatial cognitive load than to verbal cognitive load. Therefore, the tentative assumption would be in favor of domain-specific resources in WM, with asymmetry in the number of resources needed for verbal and spatial information processing, which is in accordance with previous research [18]. This premise is supported by the results of visuospatial dual-task, where no such effect was found. Therefore, the visuospatial WM component was proved to be less sensitive to cognitive load modality, which supports previous findings [28]. However, our results correspond to N. Friedman and A. Miyake’s study only with regard to the influence of verbal cognitive load on the verbal WM component. However, in contrast to existing data on domain-specific resource distribution [29], no significant distinctions were found in visuospatial task performance under visuospatial and verbal cognitive load.

Splitting the sample by the number of elements in the set yielded insight into the problem of cognitive resources distribution in WM. The results of the study indicate significant distinctions in the relative efficiency of domain-specific task performance in 4+ element sets under visuospatial and verbal cognitive load. By contrast, no such effect was revealed in 2–3 element sets. These results are in accordance with the premises of the concentric model [11] in distinguishing the region of direct access and the activated part of long-term memory regarding the domain-specific (for the activated part of long-term memory) and domain-general resources (for the region of direct access).

No significant interaction of the task type and cognitive load modality in 2–3 element trials can account for domain-general cognitive resources in the region of direct access. As no significant distinctions were observed in relative accuracy in the symmetry span and in the letter reading span task under visuospatial and verbal cognitive load, the domain-general nature of information retention in the region of direct access is assumed. The findings amplify the rationale to distinguish the region of direct access, as proposed in the concentric model [11,30], which is provided by domain-general resources. 

In 4+ element trials, significant distinctions in relative accuracy in symmetry span and in letter reading span task under visuospatial and verbal cognitive load was observed. As the number of elements exceeds the supposed capacity of the region of direct access, the task can be attributed to the activated part of long-term memory. The findings support the assumption of domain-specific resources in the activated part of long-term memory. A verbal cognitive load produced little effect on relative accuracy in the visuospatial task, while dramatically inhibiting performance in the verbal task. A visuospatial cognitive load produced a relatively stronger effect in the visuospatial task than verbal cognitive load, while in the verbal task the effect was stronger when compared with the visuospatial task. Moreover, the effect was uneven: distinctions were more significant for the verbal task than for the visuospatial task. The findings provide new insight into the asymmetry of the WM components, along with previous research emphasizing the role of verbal memory load [31] but differentiating the effect for the region of direct access and activated long-term memory.

The obtained results correspond to previous research findings of task complexity and task modality of the additional task on information retention in WM [32]. However, E. Chen and D. Bailey’s meta-analysis results account for the greater role of cognitive load complexity than cognitive load modality (which can be attributed to the specifics of arithmetic performance). Therefore, the distinctions in relative accuracy under visuospatial and verbal cognitive load found for 4+ element sets (but not for 2–3 element sets) can be due to domain-specific resources in the activated long-term memory, but not for the region of direct access. The study contributes to the debates on the matter of domain-specific and domain-general WM resources in central and peripheral WM components [14,15]. 

The question of domain-specific and domain-general resources (and, broader, unitary vs non-unitary nature of WM) is one of the substantial questions in WM research [33]. The results of our study reveal the interplay of domain-specific and domain-general resources in WM and, most significantly, demonstrates their dynamic distribution under cognitive load. An understanding of domain-specific and domain-general resources can provide insight into information presentation modalities. For instance, Cowan et al. suggest presenting information in verbal and non-verbal forms to involve both systems in the peripheral storage [15].

## 5. Conclusions

In the study, the effect of visuospatial and verbal cognitive load on visuospatial and verbal working memory task performance was established. The effect was observed only when the number of elements in the set exceeded the region of direct access capacity. The data provide new evidence in favor of domain-general resources in the region of direct access and domain-specific resources in the activated long-term memory. Another finding is the higher susceptibility of the verbal component to cognitive load, as compared with the visuospatial component.

## 6. Limitations

Performing letter reading span and symmetry span tasks concurrently with additional load tasks required substantial commitment of our subjects. While all of them were students at a linguistic university and, therefore, proficient sequential bilinguals, we attributed the fact that they managed to perform the complicated “triple” task to better working memory capacity in bilinguals (see, e.g., [34]). However, the recruiting sample at MSLU had its limitations: gender imbalance (a significantly higher number of women compared to men) was a function of the gender distribution of MSLU students.

## Figures and Tables

**Table 1:** Three-way ANOVA results for relative accuracy.

	F (1, 1253)	*p*-Value
Task type	32.4	<0.001
Load type	0.57	0.45
Items per trial	0.01	0.91
Task * Load	5.07	0.03
Task * Items	15.5	<0.001
Load * Items	4.89	0.027
Task * Load * Items	6.93	0.009

## Data Availability

All the data are available upon reasonable request to the authors.
